# Characteristics of the Dementia Care Workforce in Nursing Homes and Assisted Living Communities in the U.S.

**DOI:** 10.1093/geroni/igaf122.3894

**Published:** 2025-12-31

**Authors:** Lei Chen, Donovan Maust, Joanne Spetz

**Affiliations:** University of California, San Francisco, San Francisco, California, United States; University of Michigan, Ann Arbor, Michigan, United States; University of California, San Francisco, San Francisco, California, United States

## Abstract

The professional dementia care workforce directly supports people with Alzheimer’s disease and related dementias across multiple care settings. Although much research has focused on family and unpaid caregivers, less is known about those providing care in a professional capacity and the extent to which their characteristics influence outcomes for people with dementia. This study analyzes the first wave of the National Dementia Workforce Study (NDWS) Nursing Home (N = 394) and Assisted Living (N = 447) Staff Surveys’ Public Use Files. Weighted descriptive statistics and chi-squared tests were used to examine the overlap and distinct attributes of workers across these two settings. Findings indicate that nursing home staff were more likely to hold licenses (e.g., licensed practical/vocational nurse; certified nursing assistant), have higher education attainment, and earn higher hourly wages compared to assisted living workers. They were also more likely to report holding multiple jobs for pay and to get health insurance and benefits from their current employer. However, nursing home staff reported greater challenges in communication and more disrespectful behavior from residents’ family members. Significant differences were also observed between the two settings by country of origin and race/ethnicity. This study provides a population-level portrait of the dementia care workforce in the U.S., highlighting both commonalities and unique challenges across nursing homes and assisted living facilities. Findings can inform policy and program development to strengthen the recruitment, retention, and support of a diverse professional dementia care workforce and to improve care quality for people living with dementia.

